# Understanding healthy ageing in India: insights from multivariate regression trees

**DOI:** 10.1007/s40520-024-02815-6

**Published:** 2024-08-01

**Authors:** Ayushi Das, Preeti Dhillon

**Affiliations:** 1 International Institute for Population Sciences Deonar, Mumbai, 400088 India; 2 Department of Survey Research and Data Analytics, International Institute for Population Sciences Deonar, Deonar, Mumbai, 400088 India

**Keywords:** Healthy ageing, Multivariate regression trees, Machine learning, Older adults

## Abstract

**Background:**

Population ageing represents a significant global challenge, particularly pronounced in countries like India.

**Aims:**

This study aims to explore how factors such as socio-economic status, behaviour, and health influence healthy ageing across the Indian older population.

**Methods:**

In this study, we utilized the Longitudinal Ageing Study in India – wave 1 dataset for analysis purposes. Scores were generated for five dimensions of healthy aging, including physical, functional, mental, cognitive, and social aspects and these scores were treated as the target variables. Multivariate Regression Trees analysis was employed to identify the behavioural and socio-demographic factors associated with each dimension of healthy ageing.

**Results:**

Years of education emerge as crucial across all dimensions, positively impacting cognitive health and mitigating age-related decline in healthy ageing. Marital status, engagement in household activities, spiritual practices, and living arrangements impacts the scores of different aspects of healthy ageing. Gender disparities in healthy aging are noticeable in the 60–74 age group, with women generally having lower scores. Safety of the living environment is a crucial determinant of the mental health of the elderly across all age groups.These findings highlight the complex interplay of factors in healthy ageing outcomes.

**Conclusion:**

Our study emphasizes the pivotal role of education in fostering healthy ageing in India. Factors such as environmental safety and social participation also influence well-being. Targeted interventions addressing education, gender equality, safety, and healthcare access are vital for enhancing the ageing experience and overall well-being of older adults.

**Supplementary Information:**

The online version contains supplementary material available at 10.1007/s40520-024-02815-6.

## Introduction

The World Health Organization (WHO) launched the Decade of Healthy Ageing in 2020 to address the population ageing issues. This initiative aims to promote practices that support autonomy, productivity, and overall quality of life as people age. Healthy ageing, as defined by WHO, involves not just physical health but also mental, cognitive, and social well-being, ensuring older adults can maintain their functional abilities and well-being throughout their lives [1]. Healthy ageing typically correlates with being free from illness, injury, or pain, and is widely explored in literature through various theories. Erik Erikson’s theory of “ego integrity versus despair” from 1950 remains pivotal, focusing on how older adults assess their lives for fulfilment and satisfaction [2]. In contrast, disengagement theory posited in 1961 suggests older individuals withdraw from active life as they prepare for death [3]. Productive ageing, explored since the 1960s, emphasizes activities that contribute to society regardless of compensation, aligning with civic engagement efforts promoted by the Gerontological Society of America (GSA) [4, 5]. Cultural perspectives on ageing, such as the concept of “good old age,” vary widely, influencing views on successful ageing, which involves happiness and contentment with life, adaptation, and meaningful connections [6–8]. Successful ageing encompasses psychological and social coping mechanisms despite health challenges, diverging from definitions focusing solely on disease and disability [9, 10]. The concept of healthy ageing, originating from active ageing principles by WHO and the International Longevity Centre in 2002, emphasizes health optimization, participation, security, and lifelong learning [11]. This evolved into a broader perspective by the Swedish National Institute of Public Health, defining healthy ageing as maintaining independence and high quality of life [12]. Resilient ageing, introduced in 2014, highlights how older adults overcome challenges through coping mechanisms and enhancing their quality of life, while WHO in 2015 expanded healthy ageing to include mental, cognitive, and social dimensions for overall well-being [13, 14]. This concept was further broadened in the WHO’s ‘Decade of Healthy Ageing’ in 2019, to encompass the mental, cognitive, and social aspects of older adults [1].

A study in Korea classified healthy ageing-associated factors into physical, emotional, mental, social, and economic domains and they identified key modifiable factors for achieving healthy ageing [15]. Zaidi et al. calculated a quantitative measure of healthy ageing and compared it across the European countries [16]. Several prior studies conducted in India have investigated the association between healthy ageing and various factors [17]. Older women tend to live longer than men but often suffer from poorer health in their later years. In India, women are more likely to encounter health issues compared to men, mostly because of the social inequalities and challenges they face [18]. This study contributes to the literature by identifying the combination of factors that determine healthy ageing outcomes. Traditional regression analysis excels at estimating the effect of a single variable, but it is less capable at detecting the complex interactions between multiple variables, whereas using multivariate regression tree for this purpose is a novel way of analysis.

This study aims to elucidate the multifaceted nature of healthy ageing by investigating the interplay of various independent factors including demographic, socio-economic, and behavioural,. By comprehensively examining these factors, we seek to discern their relative contributions to different dimensions of healthy ageing, including physical, functional, mental, cognitive, and social aspects, highlighted by WHO [1]. The study further intents to identify the specific effect of each factor on these dimensions and to examine how does it varies across different age groups to understand ageing pathways which may provide valuable insights for developing strategies to improve the well-being of ageing populations.

## Methods

### Data

The data employed in this study were derived from the initial phase of the Longitudinal Study of Ageing in India (LASI wave-1), conducted from 2017 to 2018 [19]. LASI is a nationally representative longitudinal study that explores aging, health, and the socio-economic aspects of population ageing in India. The sampling methodology involved a multistage stratified area probability cluster selection. In rural areas, a three-stage sampling design was applied, while in urban areas, a four-stage sampling design was utilized during LASI wave-1. The survey encompassed a total sample of 72,250 individuals aged 45 years and above, along with their spouses, without any age restrictions. The dataset encompasses all 36 states and union territories in India. For our study, we have imposed a minimum age criterion of 45 years applying which excluded 6688 individuals from the total sample. Then, biomarker information for 5798 individuals were not available due to non-response, refusal and other reasons, and 691 observations were missing. So, after excluding these, our final sample for analysis became 59,073 individuals.

### Variable description and index creation

Healthy ageing includes multidimensional factors which contribute to the overall physical and mental wellbeing of the individual. To thoroughly examine this intricate process, we’ve considered a range of variables across different categories, broadly classified as demographic variables, socio-economic variables, and health & behavioural factors. In the demographic characteristics, gender, place of residence, marital status, years of education, and caste of the person has been taken into account. The socio-economic category includes living arrangement, wealth quintile, working status, financial support, pension, health insurance, safe environment, and ill treatment. Lastly, in the health & behavioural variables are BMI, alcohol/ tobacco consumption, food insecurity, physical activity, spiritual activity, involvement in household activity is taken. The safe environment variable is determined by assessing how safe respondents feel from crime both at home and outside, while ill-treatment is defined as any form of abuse, including physical, verbal/emotional, economic exploitation, and neglect. The details of the variables are presented in the supplementary table-1.

To investigate various facets of healthy ageing individually, five indices have been formulated: the physical health index, functional health index, mental health index, cognition index, and social index. Each index is constructed by combining specific variables sourced from the LASI and aligns with the World Health Organization’s definition of healthy ageing. Our methodology draws inspiration from a previous research article by Mandi et al., where the authors crafted a single healthy ageing index using LASI variables [16]. It’s noteworthy that our approach diverges from theirs, as we have developed five distinct indices to delve into various dimensions of healthy aging.

In constructing the physical health index, we assessed the health status of individuals, considering 9 chronic diseases: hypertension, diabetes, cancer, chronic lung disease, heart disease, stroke, bone disease, high cholesterol, and neurological disorder. The disease-free status was coded as 1, while the presence of a disease was coded as 0, penalizing the negative (diseased) physical state. Similarly, the functional health index is created by combining 13 activities of daily living variables. Those were coded as 0 if the individual was having difficulty doing them and 1 for not having difficulty. The variables were dressing, walking, bathing, eating, getting in and out of bed, toilet use, food preparation, shopping, telephone use, taking medicine, household work, managing money, and find familiar places. The mental health index comprised 10 variables that gauged respondents’ experiences, such as trouble concentrating, feelings of depression, fatigue, fear, overall satisfaction, loneliness, being bothered by things, perceiving tasks as an effort, feelings of hopefulness about the future, and happiness. Responses were recorded based on the frequency of negative thoughts (often, sometimes, mostly, or always). Cognitive ability was assessed by combining the variables: total word recall, orientation (time, month, year, day of week, place, village/town/city, landmark, district), arithmetic function (backward count, computation), executive function (paper folding, pentagons drawing), and object naming. Lastly, the social index was created by using the variables describing the social activities of the respondent and how frequently those are done i.e. daily, occasionally, rarely, or never. And the variables were how frequently does the person eat out of the house, go to park/beach for relaxing, play cards/ indoor games, play out door games/ sports/ yoga/ exercise/ jog, visits relatives/ friends, attend cultural performances/ shows/ cinema, attend religious functions, attend community/ group meetings, read books newspaper/ magazines, watch television, use a computer for email/ net surfing. All the variables used for making index is described in the supplementary Table 1 as indicators of healthy ageing. To facilitate comparisons, and for better interpretation, we standardized each variable within every index to achieve a zero mean. This involved calculating the minimum and maximum values for each variable, scaling them between 0 and 1 using the formula (var – min of var) / (max of var – min of var). Subsequently, we aggregated the scaled values of each variable sequentially as we proceeded to create each index. Finally, we normalized each index to have a zero mean. The descriptive table of age-wise mean scores of healthy ageing aspects is presented in the supplementary file.

### Empirical model

The multivariate regression tree (MRT) was first introduced by G. De’ath to predict the relationship between multiple species data with various environmental characteristics [20]. In the current paper, MRT was constructed with all five ageing indices as target variables, allowing simultaneous analysis of multiple outcome measures for a comprehensive understanding of healthy aging. The multivariate regression tree model was employed using the classification and regression trees (CART) algorithm from Scikit-learn library of Python [21]. It was employed to develop a model capable of capturing nuanced associations within the diverse set of input features from demographic, behavioural, and social input variables and their impact on the five healthy ageing indicators.

The regression tree starts with a root node that contains the entire dataset. It then splits the data into smaller groups based on certain features and values. This process continues, creating branches and decision rules at each step. The goal is to group similar data together in leaf nodes, where each leaf holds a constant value. When predicting for new data, it follows the decision rules, traversing the tree to reach a leaf and using the constant value for prediction. For example, in our dataset a variable called *wealth quintile* has 5 categories (*poorest = 0*,* poorer = 1*,* middle = 2*,* richer = 3*,* richest = 4*) then the split at 2.5 suggests that *poorest*,* poorer and middle*-income groups falling under one group whereas the *richer and richest* in another group for certain constant values of the five healthy ageing outcomes. This tree structure helps capture relationships in a way that’s easy to understand and make predictions for different subsets of the data. While partitioning, the best split based on a chosen variable and threshold is determined by the mean squared error (MSE).

Let Y_1_, Y_2_, Y_3_, Y_4_, and Y_5_ be the five outcome variables. The objective is to minimise the sum of mean squared errors (MSE) for all five outcome variables across all nodes in the tree.

$${\rm{MSE}}\; = \;\frac{1}{N}\;\Sigma _{i = 1}^N\;\Sigma _{j = 1}^5\;{({y_{ij}} - {\bar y_1})^2}$$

Where, N is the number of samples in the node, $\:{y}_{ij}$ is the actual value of the j^th^ outcome variable for the i^th^ sample in the node, and $\:\stackrel{-}{{y}_{j}}$is the mean value of the j^th^ outcome variable in the node.

When evaluating split points, the algorithm considers the combined MSE for all five outcome variables.

$$\begin{array}{l}{\rm{Weighted}}\;{\rm{MSE}}\\\; = \;\Sigma _{j = 1}^5(\frac{{{N_{left}}}}{{{N_{total}}}}\; \times \;MS{E_{left,j}}\; + \;\frac{{{N_{left}}}}{{{N_{total}}}}\;\; \times \;MS{E_{right,j}})\end{array}$$

Where, $\:{N}_{left}$, $\:{N}_{right},\:{N}_{total}$ are as defined in the previous response. $\:{MSE}_{left,\:j\:}$, $\:{MSE}_{right,\:j\:}$are the MSE values for the left and right-side nodes for the j^th^ outcome variable. The algorithm continues to recursively split nodes for all five outcome variables.

The splitting stops once the number of observations is reached to a very small size, or if the stopping criterion is achieved. Generally, before applying any machine learning model, the dataset is divided into two parts, training and testing set to test the accuracy of the models. The same to be done for the regression tree; the algorithm is applied on the training set. But there is a concern of the model overfitting the dataset only on the training set, so that when the same model is applied on the test set or any other similar dataset, then the result will not be as good. Thus, pruning is performed for regression trees, in this process a k-fold cross validation is applied to the training dataset, in which is it divided into k folds and in each run, one of the k subsets is considered as the testing set and the process is repeated over and over again for k times. This way, the optimal number for the depth of tree can be calculated and the overfitting problem can be solved.

This way the CART algorithm builds regression tree that simultaneously models relationships for all five outcome variables. CART is different from other regression models primarily because of its tree-like structure to make predictions which provides clear and interpretable way to understand the problem with an attractive visualization. In our case, the five indices of healthy ageing can be predicted using simple regression models. However, the CART algorithm offers a unique advantage by simultaneously predicting all five ageing outcomes. This approach provides a more comprehensive understanding of the potential interaction effects among the indices of healthy ageing. Additionally, the CART algorithm generates visual plots that depict the healthy ageing trajectory for individuals based on the provided input variables, offering valuable insights into the complex relationships within the ageing process.

## Results

### Descriptive results

**Fig. 1:**
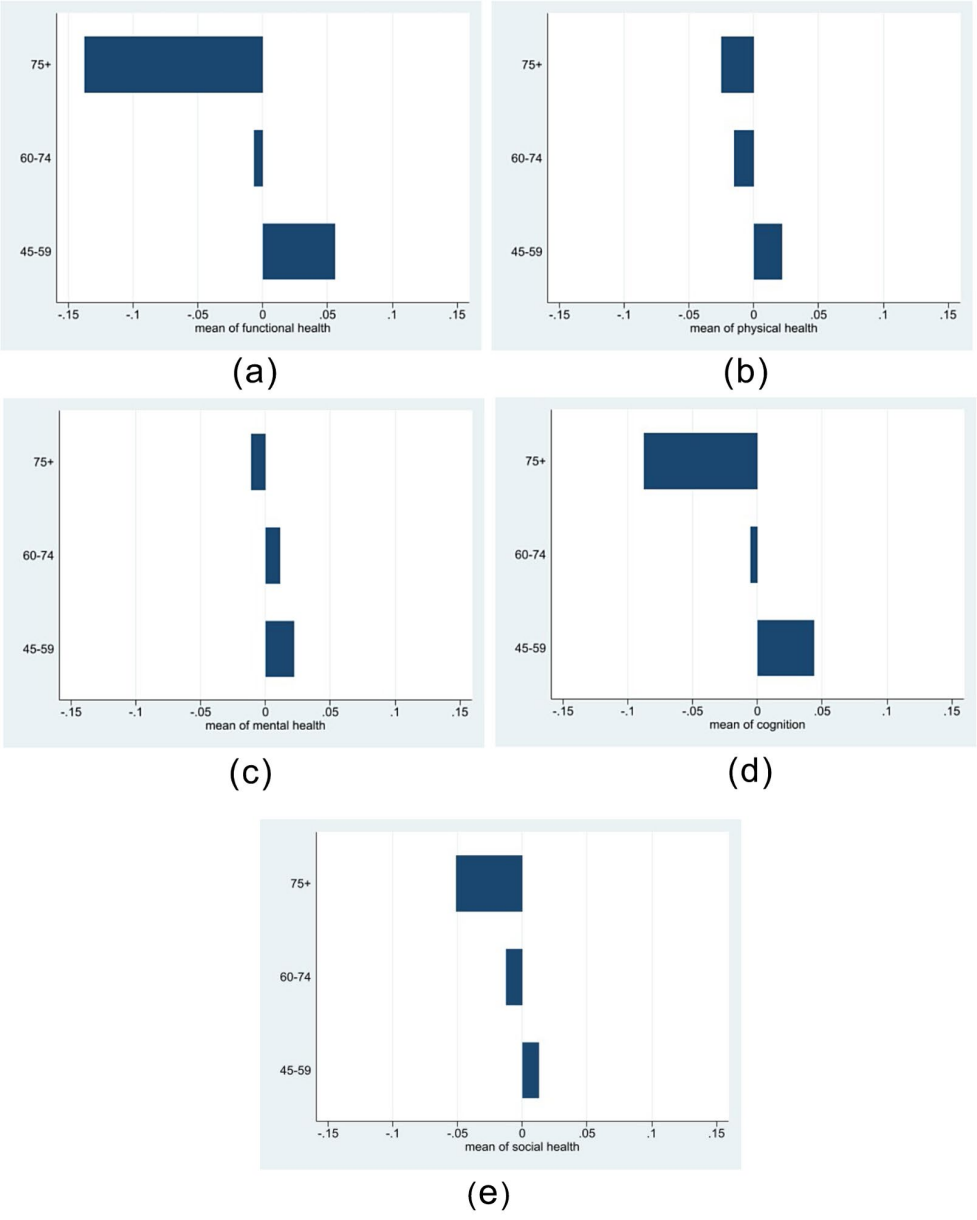
(**a**): Functional health scores. (**b**): Physical health scores. (**c**): Mental health scores. (**d**): Cognition scores. (**e**): Social scores. * Fig. 1(a, b, c, d, e) presents scores of different aspects of healthy ageing for all Indian states and Union Territories (UTs)

Figure 1 illustrates the mean scores across different age groups for five key indices of healthy aging: functional health, physical health, mental health, cognition, and social index. In the 45–59 age group, all indices show positive scores, with functional health and cognition scores notably higher, which aligns with expectations for the youngest age bracket. Moving to the 60–74 age group, we observe a shift. Physical health, cognition, and social indices exhibit negative scores, with functional health showing a slight negative trend. Remarkably, mental health remains positive, indicating relatively robust mental well-being among elderly Indians despite other health challenges. In the oldest age group (75 years and above), all indices display negative scores. Functional health and cognition scores notably lag behind the others, suggesting greater difficulties in these indices among the oldest individuals.

### Results from the MRT

The multivariate regression trees are illustrated in Fig – 2, 3, 4 & 5, depicting various paths of healthy ageing across different age groups. Each regression tree comprises 8 outputs, referred to as decision nodes, wherein the scores for the 5 healthy ageing indices are denoted as ‘value’ within each decision node. The order of presentation for each node is physical health, functional health (referred to as mobility index in most figures), mental health, cognition, and social status index, respectively. The changing colour of each node signifies the alteration in the squared error value. A darker shade of colour corresponds to a lower squared error value, indicating a more favourable result.

In all regression trees, education emerges as the predominant factor influencing the segmentation of healthy aging. Notably, education status classified as either no education or more than one year of education consistently occupies the apex position in the decision trees, indicating its pivotal role in predicting healthy ageing outcomes.

**Fig. 2:**
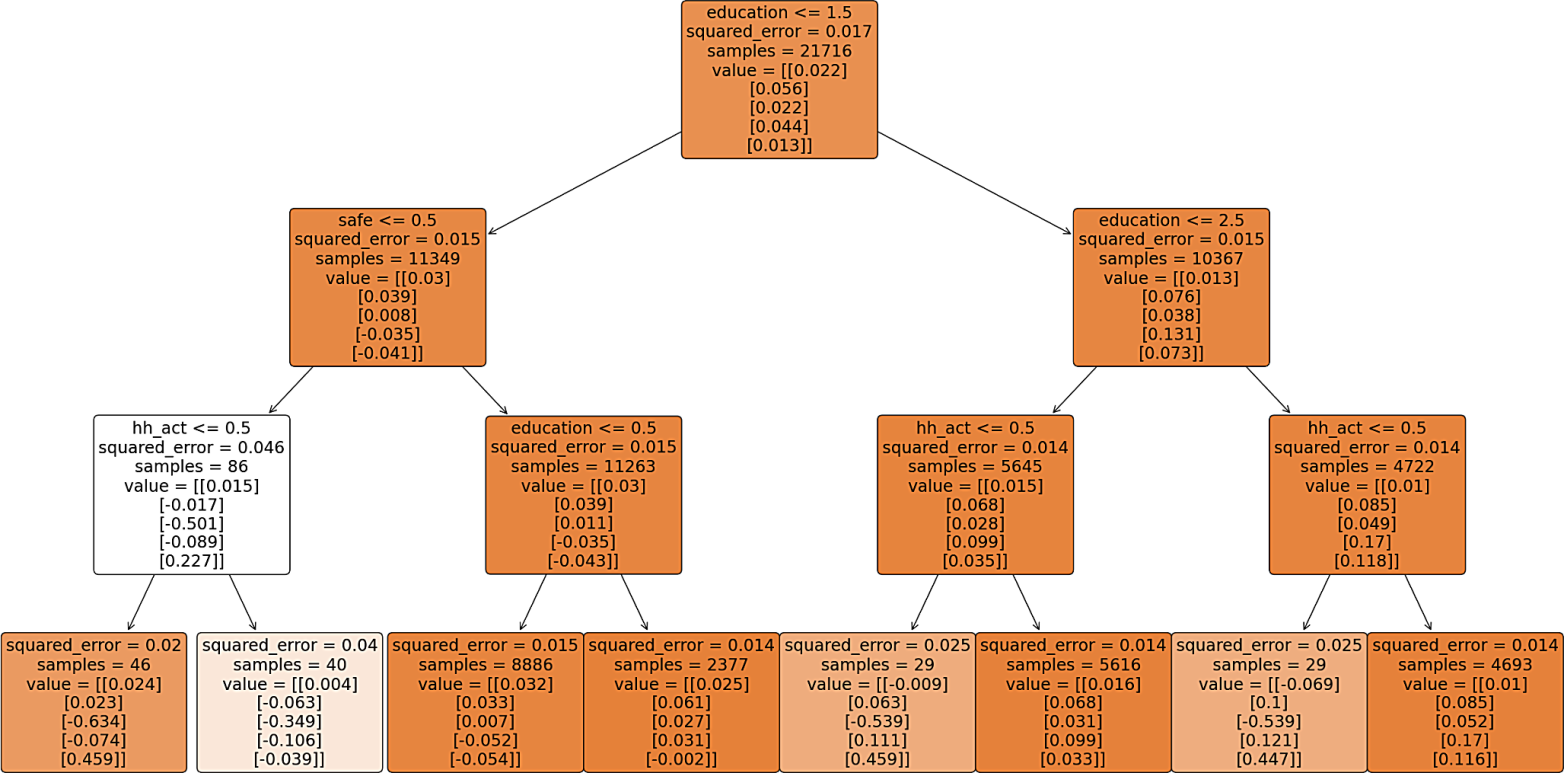
Multivariate regression tree for age group 45–59. ** variable decription: education = years of education, work_st = working status, place_resi = place of residence, safe = living in safe environment, hh_act = involvement in household activity. The categories are explain in the Supplementary table-1. The numbers < = 0.5, 1.5, 2.5 on the starting of each node is the splitting criteria of decision node

Figure 2 presents the regression tree for the 45–59 age group, highlighting two main branches based on education levels: less than 5 years and more than 5 years. On the left, individuals with less than 5 years of education, living in unsafe environments and not involved in household activities, exhibit the poorest mental health (-0.634) and cognitive (-0.074) scores, despite a high social index. Involvement in household activities further worsens their scores across all indices, with the worst-performing category showing negative functional (-0.063), mental health (-0.349), cognition (-0.106), and social index (-0.039) scores. Uneducated individuals in safer environments have slightly better social index scores (-0.054) but lower cognitive scores (-0.052) compared to their educated peers (-0.031, -0.002). On the right, cognitive scores and other indices improve with increased education. Those with more than 10 years of education are further categorized by household activity involvement, with non-participants having negative physical (-0.009, -0.069) and mental health (-0.539, -0.539) scores, while participants show positive scores across all healthy ageing indices.

**Fig. 3:**
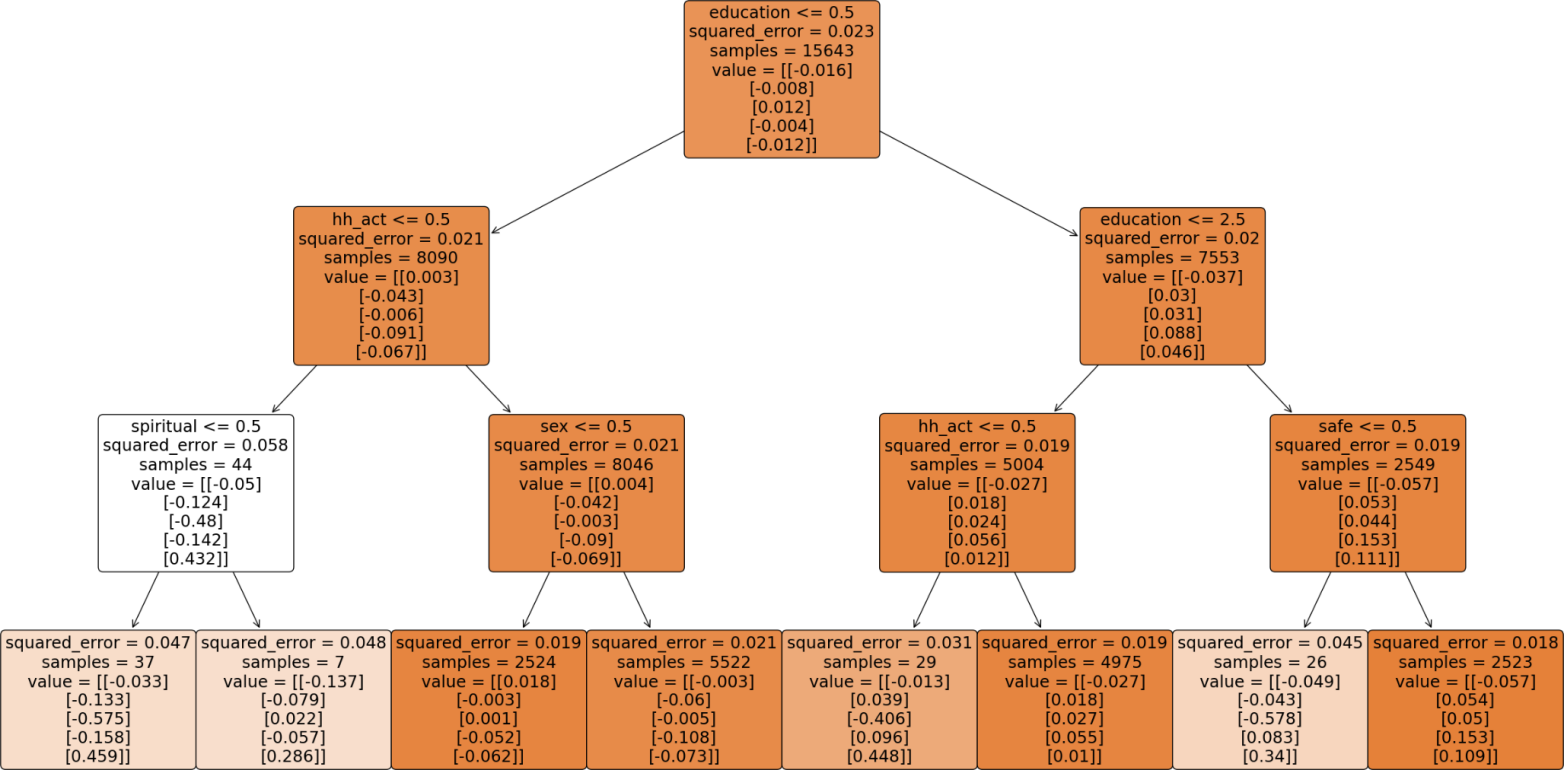
Multivariate regression tree for age group 60–74**. ** variable decription: education = years of education, work_st = working status, place_resi = place of residence, safe = living in safe environment, hh_act = involvement in household activity. The categories are explain in the Supplementary table-1. The numbers < = 0.5, 1.5, 2.5 on the starting of each node is the splitting criteria of decision node

In Fig. 3, the regression tree for the 60–74 age group also emphasizes the importance of education in determining healthy ageing scores. Individuals with more than 10 years of education and living in safer environments generally exhibit positive scores in functional (0.054), mental (0.05), cognitive (0.153), and social (0.109) health indices, though their physical health index is negative (-0.057). Physical health scores are negative across most categories in this age group, except for uneducated males involved in household activities, who have a slightly positive score (0.018). In contrast, uneducated females involved in household activities have negative scores across all indices. Those with 5 to 9 years of education involved in household activities have better mental health scores (0.027) compared to those not involved (-0.406).

**Fig. 4:**
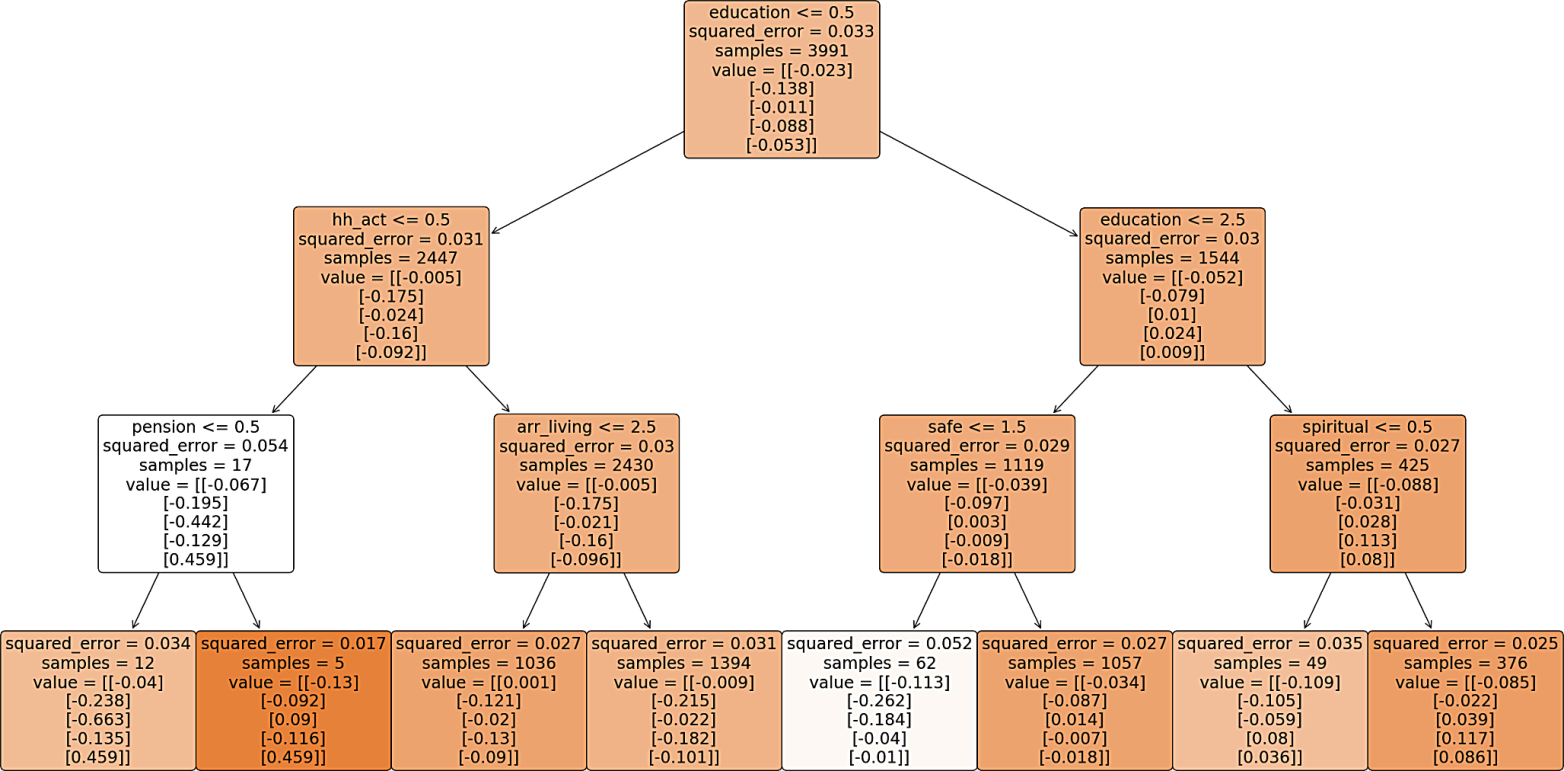
Multivariate regression tree for age group 75 and above**. ** variable decription: education = years of education, work_st = working status, place_resi = place of residence, safe = living in safe environment, hh_act = involvement in household activity. The categories are explain in the Supplementary table-1. The numbers < = 0.5, 1.5, 2.5 on the starting of each node is the splitting criteria of decision node

Figure 4 shows the regression tree for individuals aged 75 and above, highlighting key factors influencing healthy ageing scores. Education, spiritual activity, pension, and living arrangements are pivotal. Educated individuals with over 10 years of schooling and engaged in spiritual activities have the best scores, with lower physical (-0.085) and functional (-0.022) health indices but positive mental (0.039), cognitive (0.117), and social (0.086) health indices. Those not involved in spiritual activities have a negative mental health score (-0.059). A safer living environment is linked to positive mental health outcomes. Living alone or with a spouse and children results in a slightly positive physical health score (0.001). For uneducated older adults, receiving a pension positively affects their mental (0.09) and social (0.459) health. As age increases, healthy ageing scores tend to decline, shifting towards negative values.

**Fig. 5:**
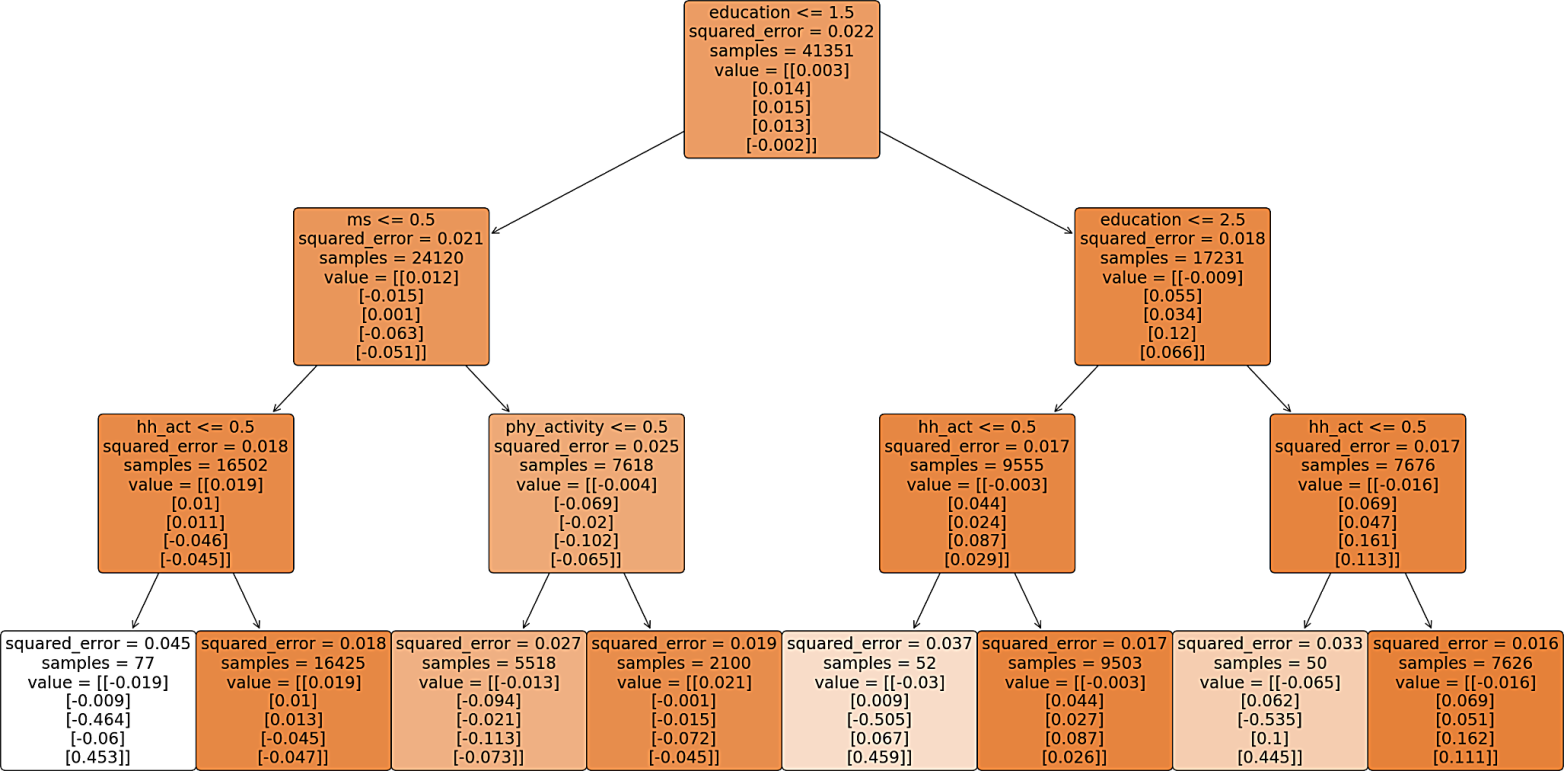
Multivariate regression tree for all age groups**. ** variable decription: education = years of education, work_st = working status, place_resi = place of residence, safe = living in safe environment, hh_act = involvement in household activity. The categories are explain in the Supplementary table-1. The numbers < = 0.5, 1.5, 2.5 on the starting of each node is the splitting criteria of decision node

Figure 5 shows the regression tree for all age groups, closely resembling the structure of Fig. 2 (45–59 age group) with a notable deviation on the leftmost side. Individuals with less than 5 years of education are categorized by marital status. Those who are still married have slightly positive healthy ageing scores, particularly if they engage in household activities, resulting in better physical (0.019), functional (0.01), and mental (0.013) health scores compared to those who do not perform household chores. Divorced or separated individuals not engaged in physical activities have negative healthy ageing scores, while those who are active have a positive physical health score (0.021). Higher education (more than 10 years) generally ensures better functional, mental, cognitive, and social health scores, although these groups tend to have negative physical health scores regardless of their activities.

In all age groups examined (45–59, 60–74, and 75 and above), education consistently emerges as a crucial determinant at the top of the decision trees. Education significantly improves scores in functional, mental, cognitive, and social health across all age groups. For ages 45–59, low education and unsafe environments lead to poorer scores, while higher education improves outcomes. In the 60–74 group, physical health scores are mostly negative, except for uneducated males involved in household activities. For those 75 and older, spiritual activity, pensions, and living arrangements positively influence scores. Overall, higher education consistently yields better scores, despite negative physical health outcomes.

## Discussion

Our analysis uncovers essential factors impacting various aspects of healthy ageing, revealing its complex nature and offering insights into how socio-economic, behavioural, and health factors interact to shape healthy aging. The findings of our analysis unveil numerous intriguing insights worth exploring. Firstly, across all age groups, years of education emerges as a significant factor influencing all dimensions of healthy ageing, which is consistent with previous literature. Long-term health differences among older individuals could result from the effect of low education, which may have impacted health from an early age and persisted into later life [22]. Healthy ageing declined over time across all educational groups, with individuals holding a college degree or higher experiencing a smaller decline compared to those without a high school diploma [23, 24]. Our findings also show that the higher level of education has a particularly pronounced positive association with cognitive health in all age groups. Wagg et al. and Avila et al. found that that the level of education impacted all cognitive index measures; with a higher educational attainment, cognitive decline is reduced [25, 26]. Higher education levels were linked to enhanced performance across diverse cognitive indices, including general mental status, episodic memory, language, and attention [27].

Secondly, our study reveals that females tend to have lower scores for healthy ageing compared to males, indicating gender disparities in healthy ageing that align with previous research [28, 29]. In the Indian context, health disparities between genders were primarily linked to factors of discrimination [30], with women having lower scores attributed to various socioeconomic and behavioural factors, such as education, household consumption quintile, and employment status [31]. The disparity in social and cognitive health in our study reveals that males consistently attain higher scores in both social functioning and cognitive abilities compared to females, irrespective of factors such as place of residence and educational attainment. The difference in individual level and social level discrimination for females in Indian society may contribute to these differences [32, 33].

The safety of the living environment is a crucial determinant of the mental health of the elderly. This is corroborated by existing literature, which highlights the substantial influence of improved housing conditions and a healthy physical and social environment on fostering positive mental health outcomes among the elderly population in India [34–36]. Contrary to our initial assumption, social participation significantly increases among the elderly residing in relatively unsafe indoor or outdoor environments, particularly among males. Although this may seem unexpected given the vulnerability of the elderly to crime or violence in such environments, the increase in social activity could be attributed to a desire to alleviate boredom. Rather than remaining at home, they may seek opportunities for social interaction to relieve mental stress [37], and engaging in religious social activities for their mental satisfaction [38]. Moreover, the growing participation of older adults in the Indian workforce, driven by the need to meet the financial expectations of their family members, leads to increased social activity. [39].

Education plays a crucial role in shaping cognitive abilities that endure into old age, though it does not appear to affect the rate of cognitive decline [40, 41]. Enhancing early-life conditions holds promise for improving cognitive function in adulthood and alleviating public health challenges linked to cognitive ageing and dementia. Importantly, while education initially benefits cognitive functioning in older adults, its positive effects diminish as cognitive impairment worsens. This underscores the complex interplay between education, ageing, and cognitive health in later life [42].

This study has a few limitations. Firstly, the regression tree method is intended for identifying patterns in data rather than establishing causal relationships. Secondly, the results presented in figures are based on group averages, and individual preferences for certain behavioural factors may influence outcomes. However, since the survey used is nationally representative and ageing outcomes are not selectively biased, individual differences are likely averaged out. Lastly, limited variability in the variables may also impact our findings, particularly as multivariable regression methods are commonly used in research with count measures as outcome variables.

## Conclusion

In conclusion, our study sheds light on various factors influencing healthy ageing among older adults in India. Education emerges as a significant predictor across all dimensions of healthy ageing, highlighting its role in promoting cognitive health and mitigating age-related decline. Gender disparities in healthy ageing are evident, with females generally exhibiting lower scores, possibly influenced by societal discrimination and factors. The safety of the living environment and social participation also play crucial roles in the mental well-being of the elderly, with social activities potentially serving as coping mechanisms in unfavourable environments. Moreover, factors such as marital status, engagement in household activities, spiritual practices, and living arrangements further influence healthy ageing scores. These findings underscore the need for targeted interventions addressing education, gender equality, environmental safety, and healthcare access to promote healthy ageing and enhance the overall well-being of older adults in India. Continued research and policy efforts in these areas are essential for addressing the evolving needs of the ageing population and ensuring a healthier and more equitable ageing experience for all.

### Electronic supplementary material

Below is the link to the electronic supplementary material.

Supplementary Material 1

## Data Availability

No datasets were generated or analysed during the current study.

## References

[1] World Health Organization (2019) Decade of Healthy Ageing 2020–2030

[2] Erikson EH (1950) Childhood and society. Childhood and society. Brill Schöningh, New york, pp 116–118

[3] Cumming E, Henry WE (1962) Growing Old: the process of disengagement. Soc Work 7:122

[4] Butler RN, Oberlink MR, Schechter M, Nihon Senpaku Shinkōkai (1990) The promise of productive aging: from biology to social policy. (No Title)

[5] Martinson M, Minkler M (2006) Civic Engagement and older adults: a critical perspective. Gerontologist 46:318–324 16731870 10.1093/geront/46.3.318

[6] Jeste DV, Depp CA, Vahia IV (2010) Successful cognitive and emotional aging. World Psychiatry 9:78–84 20671889 10.1002/j.2051-5545.2010.tb00277.x PMC2912035

[7] Havighurst RJ (1961) Successful aging. Gerontologist 1:8–13 10.1093/geront/1.1.8

[8] Rowe JW, Kahn RL (1987) Human aging: usual and successful. Science 237:143–149 3299702 10.1126/science.3299702

[9] Neugarten BL (1972) Personality and the aging process. Gerontologist 12:9–15 5028195 10.1093/geront/12.1_Part_1.9

[10] Young Y, Frick KD, Phelan EA (2009) Can successful aging and chronic illness coexist in the same individual? A Multidimensional Concept of successful aging. J Am Med Dir Assoc 10:87–92 19187875 10.1016/j.jamda.2008.11.003

[11] WHO (2002) Active ageing: a Policy Framework. Age-Friendly World

[12] The Swedish National Institute of Public Health (2006) Healthy Ageing- A Challenge for Europe

[13] Hicks MM, Conner NE (2014) Resilient ageing: a concept analysis. J Adv Nurs 70:744–755 23919385 10.1111/jan.12226

[14] WHO (2015) World Report on Ageing and Health

[15] A SYSTEMATIC REVIEW ON FACTORS INFLUENCING THE HEALTHY AGING: A KOREAN PERSPECTIVE. https://www.researchgate.net/publication/356849502. Accessed 17 Jun 2024

[16] Zaidi A, Gasior K, Zolyomi E, Schmidt A, Rodrigues R, Marin B (2017) Measuring active and healthy ageing in Europe. J Eur Social Policy 27:138–157 10.1177/0958928716676550

[17] Mandi R, Bansod DW, Goyal AK (2023) Exploring the association of lifestyle behaviors and healthy ageing among the older adults in India: evidence from LASI survey. BMC Geriatr 23:675 37853323 10.1186/s12877-023-04367-2 PMC10585826

[18] Das S, Prasad J (2023) Gender differences in determinants of the components of the Frailty phenotype among older adults in India: findings from LASI Wave-1. Int J Environ Res Public Health 20:3055 36833748 10.3390/ijerph20043055 PMC9965095

[19] LASI (2020) Longitudinal Ageing Study in India (LASI) Wave 1, 2017-18, India Report. https://iipsindia.ac.in/lasi

[20] MULTIVARIATE REGRESSION TREES: A NEW TECHNIQUE, FOR MODELING SPECIES–ENVIRONMENT RELATIONSHIPS - De’ath – 2002 - Ecology - Wiley Online Library. https://esajournals.onlinelibrary.wiley.com/doi/full/10.1890/0012-9658%282002%29083%5B1105%3AMRTANT%5D2.0.CO%3B2. Accessed 15 Jun 2024

[21] Pedregosa et al (2011) Scikit-learn. Machine Learning in Python

[22] Wu Y-T, Daskalopoulou C, Terrera GM et al (2020) Education and wealth inequalities in healthy ageing in eight harmonised cohorts in the ATHLOS consortium: a population-based study. Lancet Public Health 5:e386–e394 32619540 10.1016/S2468-2667(20)30077-3 PMC7739372

[23] McLaughlin SJ, Kim S, Li LW, Zhang J (2020) Educational differences in trajectories and determinants of healthy ageing in midlife and older americans. Maturitas 134:21–28 32143772 10.1016/j.maturitas.2020.01.002

[24] Wagg E, Blyth FM, Cumming RG, Khalatbari-Soltani S (2021) Socioeconomic position and healthy ageing: a systematic review of cross-sectional and longitudinal studies. Ageing Res Rev 69:101365 34004378 10.1016/j.arr.2021.101365

[25] Avila R, Moscoso MAA, Ribeiz S, Arrais J, Jaluul O, Bottino CMC (2009) Influence of education and depressive symptoms on cognitive function in the elderly. Int Psychogeriatr 21:560–567 19327202 10.1017/S1041610209008928

[26] Okamoto S (2019) Socioeconomic factors and the risk of cognitive decline among the elderly population in Japan. Int J Geriatr Psychiatry 34:265–271 30370551 10.1002/gps.5015

[27] Chen Y, Qi D, Qin T et al (2019) Brain Network Connectivity mediates Education-related cognitive performance in healthy Elderly adults. Curr Alzheimer Res 16:19–28 30345918 10.2174/1567205015666181022094158

[28] Schladitz K, Förster F, Wagner M, Heser K, König H-H, Hajek A, Wiese B, Pabst A, Riedel-Heller SG, Löbner M (2022) Gender specifics of healthy ageing in older age as seen by women and men (70+): a Focus Group Study. Int J Environ Res Public Health 19:3137 35270831 10.3390/ijerph19053137 PMC8909956

[29] Pengpid S, Peltzer K (2021) Ethnic and gender disparities in healthy ageing among people 50 years and older in South Africa. Geriatr (Basel) 6:79 10.3390/geriatrics6030079 PMC8395815 34449634

[30] Irshad CV, Dash U (2021) Healthy Ageing and Gender Gap in India; Evidence from the Longitudinal Ageing Study in India -Wave 1

[31] Srivastava S, Muhammad T, Paul R, Khan KA (2023) Multivariate decomposition of gender differentials in successful aging among older adults in India. BMC Geriatr 23:59 36721109 10.1186/s12877-023-03753-0 PMC9890860

[32] Kulkarni S, Gade A, Shah S, Khese S (2023) Gender Differences in Social Participation and Cognitive Health in Ageing indians. J Indian Acad Geriatr 19:p176

[33] Singh PK, Jasilionis D, Oksuzyan A (2018) Gender difference in cognitive health among older Indian adults: a cross-sectional multilevel analysis. SSM - Popul Health 5:180–187 30073185 10.1016/j.ssmph.2018.06.008 PMC6068074

[34] Firdaus G (2017) Built Environment and Health outcomes: identification of contextual risk factors for Mental Well-being of older adults. Ageing Int 42:62–77 10.1007/s12126-016-9276-0

[35] Muhammad T, Meher T, Sekher TV (2021) Association of elder abuse, crime victimhood and perceived neighbourhood safety with major depression among older adults in India: a cross-sectional study using data from the LASI baseline survey (2017–2018). BMJ Open 11:e055625 34907072 10.1136/bmjopen-2021-055625 PMC8671981

[36] Srivastava S (2021) Geriatric Mental Health issues from India. In: Shankardass MK (ed) Ageing issues in India: practices, perspectives and policies. Springer, Singapore, pp 301–309

[37] Kar B (2017) Factors affecting quality of life of older persons-a qualitative study from Bhubaneswar, India. J Geriatric Care Res 2017:47–54

[38] Gautam R, Saito T, Kai I (2007) Leisure and religious activity participation and mental health: gender analysis of older adults in Nepal. BMC Public Health 7:299 17953749 10.1186/1471-2458-7-299 PMC2140059

[39] Ramachandran M, D’Souza SA (2016) A cross-sectional survey on older adults’ community mobility in an Indian Metropolis. J Cross Cult Gerontol 31:19–33 26706252 10.1007/s10823-015-9276-7

[40] Lövdén M, Fratiglioni L, Glymour MM, Lindenberger U, Tucker-Drob EM (2020) Education and cognitive functioning across the Life Span. Psychol Sci Public Interest 21:6–41 32772803 10.1177/1529100620920576 PMC7425377

[41] Frontiers | Positive Effects of Education on Cognitive Functioning Depend on Clinical Status and, Severity N https://www.frontiersin.org/articles/10.3389/fnhum.2021.723728/full. Accessed 17 Jun 2024 10.3389/fnhum.2021.723728 PMC8459869 34566608

[42] Parisi J, Rebok G, Xue Q-L, Fried L, Seeman T, Tanner E, Gruenewald T, Frick K, Carlson M (2012) The role of Education and Intellectual Activity on Cognition. J Aging Res 2012:416132 22928110 10.1155/2012/416132 PMC3423895

