# Supplementary material for: Understanding healthy ageing in India: insights from multivariate regression trees
Source: Aging Clin Exp Res. 2024 Aug 1;36(1):158. doi: 10.1007/s40520-024-02815-6 (PMC11294380; doi:10.1007/s40520-024-02815-6)
Supplement: Supplementary file 1 — Supplementary Material 1 [file 40520_2024_2815_MOESM1_ESM.docx]

**Supplementary table 1: Determinants and indicators of healthy ageing**

| *Determinants of healthy ageing* | |
| --- | --- |
| **Variable name** | **Categories** |
| Sex | 0 = male, 1 = female |
| Place of residence | 0 = rural, 1 = urban |
| Marital status | 0 = currently married, 1 = widowed, 2 = divorced/separated/deserted/others |
| Education | 0 = no schooling, 1 = less than 5 years, 2 = 5-9 years completed, 3 = 10 years or more |
| Caste | 0 = others, 1 = SC, 2 = ST, 2 = OBC |
| Living arrangement | 0 = living alone, 1 = living with spouse and/or others, 2 = living with spouse and children, 3 = living with children and others, 4 = living with others only |
| Safe environment | 0 = not safe at all, 1 = not very safe, 2 = safe, 3 = completely safe |
| Ill treated | 0 = not ill treated, 1 = ill treated |
| MPCE quintile | 0 = poorest, 1 = poorer, 32 = middle, 3 = richer, 4 = richest |
| Working status | 0 = never worked, 1 = currently working, 2 = worked previously currently not |
| Financial support receiving | 0 = no, 1 = yes |
| Pension | 0 = not receiving, 1 = receiving/ expected |
| Health insurance | 0 = no, 1 = yes |
| BMI | 0 = underweight, 1= normal, 2=overweight/obese |
| Alcohol/tobacco consumption | 0 = not consumed, 1 = consumed |
| Food security | 0 = not compromised, 1 = compromised |
| Vigorous physical activity | 0 = never, 1 = sometimes, 2 = daily |
| Spiritual activity | 0 = never, 1 = occasionally, 2 = once in a week, 3 = some days in week, 4 = everyday |
| Household activity | 0 = not involved, 1 = involved |
|  |  |
| *Indicators of healthy ageing* | |
| **Aspect of healthy ageing** | **Variables included** |
| Physical | Hypertension, Diabetes, Cancer, Chronic lung disease, Heart disease, Stroke, Bone disease, High cholesterol, and Neurological disorder  (categories = have disease, No disease) |
| Functional | Dressing, Walking, Bathing, Eating, Getting in and out of bed, Toilet use, Food preparation, Shopping, Telephone use, Taking medicine, Household work, Managing money, and Find familiar places (categories = have difficulty, do not have difficulty) |
| Mental | Trouble concentrating, Feelings of depression, Fatigue, Fear, Overall satisfaction, Loneliness, Being bothered by things, Perceiving tasks as an effort, Feelings of hopefulness about the future, and Happiness (categories = often, sometimes, mostly, or always) |
| Cognitive | Total word recall, orientation (time, month, year, day of week, place, village/town/city, landmark, district), arithmetic function (backward count, computation), executive function (paper folding, pentagons drawing), object naming |
| Social | How frequently does the person Eat out of the house, Go to park/beach for relaxing, play cards/ indoor games, Play out door games/ sports/ yoga/ exercise/ jog, Visits relatives/ friends, Attend cultural performances/ shows/ cinema, Attend religious functions, Attend community/ group meetings, Read books newspaper/ magazines, Watch television, Use a computer for email/ net surfing (categories = daily, occasionally, rarely, or never) |

|  |  | **Age groups** | | |
| --- | --- | --- | --- | --- |
|  |  | 45-59 | 60-74 | 75 above |
| **Aspects of healthy ageing** | Functional health | 0.056 | -0.006 | -0.138 |
|  | Physical health | 0.022 | -0.015 | -0.025 |
|  | Mental health | 0.022 | 0.011 | -0.011 |
|  | Cognition | 0.044 | -0.005 | -0.087 |
|  | Social | 0.013 | -0.013 | -0.051 |

**Supplementary table 2**: **Descriptive table of age-wise mean scores of healthy ageing aspects**

**Supplementary table 3**: **Number of participants from the different states and UTs**

| **States and UTs** | **Number of samples** |
| --- | --- |
| Uttar Pradesh | 3,876 |
| Bihar | 3,175 |
| West Bengal | 3,052 |
| Maharashtra | 3,028 |
| Tamil Nadu | 2,958 |
| Madhya Pradesh | 2,434 |
| Odisha | 2,356 |
| Kerala | 2,078 |
| Jharkhand | 2,075 |
| Andhra Pradesh | 1,972 |
| Rajasthan | 1,966 |
| Telangana | 1,927 |
| Gujarat | 1,898 |
| Karnataka | 1,893 |
| Punjab | 1,821 |
| Assam | 1,792 |
| Chhattisgarh | 1,782 |
| Haryana | 1,554 |
| Jammu & Kashmir | 1,275 |
| Himachal Pradesh | 1,183 |
| Uttarakhand | 1,182 |
| Puducherry | 1,155 |
| Delhi | 1,125 |
| Nagaland | 1,118 |
| Goa | 1,117 |
| Manipur | 1,080 |
| Andaman & Nicobar Islands | 1,017 |
| Mizoram | 1,015 |
| Lakshadweep | 993 |
| Tripura | 953 |
| Arunachal Pradesh | 917 |
| Dadra & Nagar Haveli | 896 |
| Daman & Diu | 825 |
| Meghalaya | 809 |
| Chandigarh | 776 |
| **Total** | **59,073** |
